# Testosterone Therapy Does Not Affect Coagulation in Male Hypogonadism: A Longitudinal Study Based on Thrombin Generation

**DOI:** 10.1210/clinem/dgae317

**Published:** 2024-05-08

**Authors:** Valeria Lanzi, Rita Indirli, Armando Tripodi, Marigrazia Clerici, Marco Bonomi, Biagio Cangiano, Iulia Petria, Maura Arosio, Giovanna Mantovani, Emanuele Ferrante

**Affiliations:** Endocrinology Unit, Fondazione IRCCS Ca’ Granda Ospedale Maggiore Policlinico, 20122 Milan, Italy; Department of Clinical Sciences and Community Health, University of Milan, 20122 Milan, Italy; Endocrinology Unit, Fondazione IRCCS Ca’ Granda Ospedale Maggiore Policlinico, 20122 Milan, Italy; Department of Clinical Sciences and Community Health, University of Milan, 20122 Milan, Italy; Angelo Bianchi Bonomi Hemophilia and Thrombosis Center, Fondazione IRCCS Ca’ Granda Ospedale Maggiore Policlinico, 20122 Milan, Italy; Fondazione Luigi Villa, 20122 Milan, Italy; Angelo Bianchi Bonomi Hemophilia and Thrombosis Center, Fondazione IRCCS Ca’ Granda Ospedale Maggiore Policlinico, 20122 Milan, Italy; Department of Endocrine and Metabolic Diseases, IRCCS Istituto Auxologico Italiano, 20149 Milan, Italy; Department of Medical Biotechnologies and Translational Medicine, University of Milan, 20133 Milan, Italy; Department of Endocrine and Metabolic Diseases, IRCCS Istituto Auxologico Italiano, 20149 Milan, Italy; Department of Medical Biotechnologies and Translational Medicine, University of Milan, 20133 Milan, Italy; Endocrinology Unit, Fondazione IRCCS Ca’ Granda Ospedale Maggiore Policlinico, 20122 Milan, Italy; Department of Clinical Sciences and Community Health, University of Milan, 20122 Milan, Italy; Endocrinology Unit, Fondazione IRCCS Ca’ Granda Ospedale Maggiore Policlinico, 20122 Milan, Italy; Department of Clinical Sciences and Community Health, University of Milan, 20122 Milan, Italy; Endocrinology Unit, Fondazione IRCCS Ca’ Granda Ospedale Maggiore Policlinico, 20122 Milan, Italy; Department of Clinical Sciences and Community Health, University of Milan, 20122 Milan, Italy; Endocrinology Unit, Fondazione IRCCS Ca’ Granda Ospedale Maggiore Policlinico, 20122 Milan, Italy

**Keywords:** male hypogonadism, testosterone, thrombin generation assay, coagulation, thrombosis

## Abstract

**Context:**

Testosterone therapy has been variably associated with increased thrombotic risk but investigations of global coagulation in this setting are lacking.

**Objective:**

This work aimed to compare global coagulation of hypogonadal men before (T0) and 6 months after (T1) starting testosterone replacement therapy (TRT), and healthy controls (HCs).

**Methods:**

An observational prospective cohort study was conducted at 2 tertiary endocrinological ambulatory care centers. Patients included 38 men with hypogonadism (mean age 55 years, SD 13) and 38 age-matched HCs. Thrombin generation assay (TGA) was performed at T0 and T1 in hypogonadal men and in HCs. TGA is an in vitro procedure based on the continuous registration of thrombin generation and decay under conditions mimicking the process that occurs in vivo. The following TGA parameters were recorded: lag time; thrombin-peak concentration; time-to-reach peak, velocity index, and endogenous thrombin potential (ETP), the latter representing the total amount of thrombin generated under the driving forces of procoagulants opposed by the anticoagulants. Protein C, antithrombin, factor (F) VIII, and fibrinogen were assessed.

**Results:**

No changes in TGA parameters were observed between T0 and T1. Hypogonadal men displayed significantly higher ETP, fibrinogen, and significantly lower antithrombin levels both at T0 and T1 compared to HCs. Thrombin peak of hypogonadal men was significantly higher than HCs at T0 but not at T1. ETP and antithrombin were correlated with testosterone levels.

**Conclusion:**

Hypogonadal men display a procoagulant imbalance detected by increased thrombin generation. Short-term TRT does not worsen global coagulation, suggesting that the treatment can be safely prescribed to men diagnosed with hypogonadism.

Venous thromboembolism (VTE) is a multifactorial disease resulting from hemodynamic changes such as reduction of blood flow or turbulence, endothelial injury or dysfunction, and blood hypercoagulability (1). Circumstantial risk factors that may influence VTE risk are recent surgery, cancer, and prolonged immobilization, with sex hormones advocated as additional pathogenetic factors (2).

An increased risk of thromboembolic events has been reported in association with oral contraceptive pills and hormonal replacement therapy in women (3-5). However, the role of endogenous testosterone and testosterone replacement therapy (TRT) in men remains controversial.

Testosterone, at physiological concentrations, has a beneficial influence on the hemostatic system as measured in vitro. In fact, both testosterone and its 5 alpha-reduced derivative dihydrotestosterone exert an inhibitory effect on primary hemostasis, by preventing adenosine diphosphate–mediated platelet aggregation (6-8). Moreover, testosterone has an inhibitory effect on coagulation and promotes fibrinolysis (9). Consistently, low testosterone levels have been correlated with increased platelet activity and a procoagulant profile (10-13). Current evidence, however, has failed to provide an association between endogenous testosterone levels in the lower quartile of normal range and new incident cases of VTE compared to individuals with testosterone levels in the middle or upper quartiles (14, 15).

It is well known that TRT leads to an increase of hematocrit, blood viscosity (16), and estrogen circulating levels (17), all factors that may potentially influence VTE risk. Four population studies reported an association between testosterone therapy and thrombotic risk (18-21). Interestingly, studies by Martinez et al and Walker et al showed that this association reached a peak within 6 months since the start of treatment (18, 19). Nevertheless, 3 meta-analyses of randomized controlled trials did not find an increased risk of VTE associated with TRT compared to placebo (22-24).

With this gap of knowledge, we aimed to investigate the variation in the global coagulation profile of hypogonadal men before and 6 months after starting TRT. Secondary aims were to compare the global coagulation profile of hypogonadal men with healthy controls (HCs), and to assess the association of coagulation with hormonal and metabolic variables. Coagulation was assessed by thrombin generation assay (TGA), an in vitro procedure based on the continuous registration of thrombin generation (mediated by procoagulants) and decay (mediated by anticoagulants).

Compared to traditional coagulation tests such as the prothrombin time, activated partial thromboplastin time, or viscoelastometry, which hardly reflect the complex and integrated mechanisms of coagulation, TGA can be considered as the closest approximation to the process occurring in vivo (25). In fact, coagulation in TGA is activated by much smaller amounts of tissue factor and phospholipids than those used in other coagulation tests (26). Furthermore, the formulations of traditional tests do not include thrombomodulin, the physiological protein C (PC) activator; hence PC in those tests cannot be optimally activated to represent what occurs in vivo. A growing body of evidence has demonstrated the ability of TGA to predict the risk of first and recurrent thrombotic events (27-29).

## Materials and Methods

### Study Design and Procedures

We conducted a multicenter, observational prospective cohort study to assess the effects of short-term TRT on the global coagulation profile of hypogonadal men.

Patients were selected among those followed up at 2 tertiary endocrinological units in Milan, Northern Italy (Fondazione IRCCS Ca’ Granda Ospedale Maggiore Policlinico and IRCCS Istituto Auxologico Italiano). Male hypogonadism was defined by reduced levels of total and/or calculated free testosterone (<12.0 nmol/L and <220 pmol/L, respectively) in men with sexual dysfunction (ie, erectile dysfunction, decreased libido, reduced nocturnal/morning erections), according to current guidelines (30). Clinical history was collected and radiological investigations and further hormonal assessments (in particular, luteinizing hormone and follicle-stimulating hormone) were carried out as appropriate to differentiate between functional hypogonadism, organic primary, and secondary hypogonadism (30). Adult patients (aged ≥18 years) with newly diagnosed hypogonadism and who had not received treatment with testosterone or gonadotrophins were included. Patients with known hereditary coagulation disorders, Klinefelter syndrome (1), or on anticoagulation (parenteral or oral) treatment, were excluded. Among patients with secondary hypogonadism, only those without pituitary hormone excess, and without uncompensated pituitary hormone deficiencies, were selected.

Patients' evaluations were scheduled before and 6 months after starting TRT (T0 and T1, respectively). Blood samples were collected for assessment of the coagulation profile.

Patients received transdermal testosterone 2% gel on a daily basis, or long-acting injectable testosterone undecanoate, which was administered at baseline, after 6 weeks (loading dose), and then every 12 weeks.

Information on smoking habit, arterial hypertension, dyslipidemia, diabetes mellitus, thromboembolic events, body mass index (BMI), fasting plasma glucose, total cholesterol, triglycerides, low-density lipoprotein (LDL) cholesterol, total testosterone, complete blood count, and prostate-specific antigen (PSA) were extracted from hospital records. Diagnostic delay was arbitrarily estimated as the time period between self-reported sexual symptom onset and start of TRT.

HCs were recruited among male medical students and hospital staff. They were matched by age (±5 years) to the patient population and were free from current and past thrombotic events, anticoagulant drugs, or coagulation disorders known to affect TGA.

All study procedures were in accordance with the principles set out in the Declaration of Helsinki. The study was approved by the Milan Area 2 ethics committee (approval ID 396). Written informed consent was obtained from all individuals included in the study.

### Blood Sampling and Plasma Preparation

Blood was collected from an antecubital vein into vacuum tubes containing one-tenth volume of trisodium citrate 109 mM (Becton Dickinson). For hypogonadal patients receiving TRT, blood samples were taken 2 hours after application of testosterone transdermal gel, or at the end of the dosing interval in case of injectable long-acting testosterone undecanoate (31). Little variability in plasma testosterone concentrations is expected during treatment with either formulation (32, 33). Nevertheless, this sampling schedule was established in accordance with current guidelines on testosterone treatment monitoring (31).

Citrated whole blood was centrifuged for 20 minutes (controlled room temperature) at 2880*g* to prepare platelet-poor plasma that was aliquoted in plastic-capped tubes, quickly frozen by immersion in liquid nitrogen, and stored at −70 °C until testing. To limit between-assay variability, an equal number of samples from patients and HCs were tested in the same run. All the experimental procedures were conducted at the Angelo Bianchi Bonomi Hemophilia and Thrombosis Center, Ospedale Maggiore Policlinico, Milan, Italy.

### Thrombin Generation Assay

TGA was assessed according to Hemker et al (34) with a homemade method as described previously (35). Testing was based on the activation of coagulation after addition to plasma of small amounts of human recombinant relipidated tissue factor (rTF, 1 pM) (Recombiplastin 2G, Werfen) and synthetic phospholipids (PL, 1.0 μM) (Avanti Polar) as coagulation triggers. Testing was performed with the addition of soluble rabbit thrombomodulin (Haematologic Technologies) (2 nM). Thrombomodulin is the physiological activator of PC and is located on endothelial cells (25). Registration of thrombin generation was obtained with a fluorogenic substrate (Z-GlyGly-Arg-AMC HCl, Bachem) (617 μM) by means of a dedicated fluorometer (Fluoroskan Ascent, Thermo LabSystems). The readings were recorded and analyzed with dedicated software (Thrombinoscope, Thrombinoscope BV), which displays the curve of thrombin concentration as a function of time and calculates the following parameters: the time (minutes) between the addition of the triggers and the initiation of thrombin generation (lag time); the thrombin peak (nM); the time (minutes) needed to reach the peak (time to peak); the area under the curve, defined as endogenous thrombin potential (ETP) and expressed as nM × min; and the velocity index, defined as [peak/(TT peak—lag time)] and expressed as nM/min.

### Other Coagulation Parameters

PC and antithrombin were measured as chromogenic activity by means of commercial kits (Hemosil antithrombin and Hemosil PC; Werfen). Factor (F) VIII, and fibrinogen were measured as described (36). FVIII results were reported as percentage activity relative to pooled normal plasma with an (arbitrary) activity of 100%.

### Hormonal Assay

Circulating total testosterone concentrations were assessed by an Elecsys Testosterone II (Calibrator reference: 05200067190) test marketed by Roche Diagnostics (RRID:AB_2783736). This method is standardized via isotope dilution–gas chromatography/mass spectrometry. The assay has a lower limit of detection of 0.087 nmol/L, a functional sensitivity of 0.4 nmol/L, and interassay or intra-assay coefficients of variation of less than 5%.

### End Points

The primary end point was the variation in the global coagulation profile of hypogonadal men from baseline to 6-month TRT, as defined by TGA and other coagulation parameters.

Secondary end points were the comparison of the coagulation profile of hypogonadal men with HCs and the association of coagulation parameters with hormonal and metabolic variables.

### Statistical Analysis

Distribution of quantitative variables was assessed by Shapiro-Wilk test. Normally distributed quantitative variables were expressed as mean and SD, whereas variables with a skewed distribution were reported as median and interquartile range (IQR) or minimum to maximum range; qualitative variables were represented as absolute frequencies. Paired or unpaired *t* test was performed to compare means of normally distributed variables. Alternatively, the nonparametric Mann-Whitney and Wilcoxon tests were used. Comparisons among 3 groups were performed by Kruskal-Wallis test. Fisher exact test or chi-square test were employed to compare frequencies of qualitative variables between 2 or more groups. Bonferroni correction for multiple comparisons was applied where indicated. Univariate association analysis was performed by Pearson correlation test or Spearman rank correlation test as appropriate. Correction for covariates was performed by analysis of covariance.

A 2-sided *P* value was considered statistically significant when less than.05. Analysis was performed with GraphPad Prism (version 10) and IBM SPSS Statistics (version 29).

### Power Analysis

For power analysis, we used ETP as a reference parameter.

Given that no data about TGA in individuals affected with hypogonadism are available, we considered our previously published data on TGA in a group of normal participants compared to patients with Cushing syndrome, a condition proven to be related to hypercoagulability (37). In that study, normal participants showed a mean (SD) ETP value of 902 (222) nM/min, while in Cushing's syndrome patients ETP was 1284 (353) nM/min (mean difference: 302 (439) nM/min). Since current evidence on coagulative derangements in male hypogonadism is inconsistent, we assumed hypogonadal men at baseline to be similar to normal individuals.

For the primary outcome, using a *t* test for dependent samples, a sample size of 25 participants is required to reject the null hypothesis that the mean ETP is not different before and after treatment, with a power of 90% and a probability of type I error of 5%.

For the secondary outcome, a 1:1 ratio between the 2 groups (hypogonadal patients and HCs) was set. Assuming a mean ETP of 982 (222) nM/min in the control group and of 1284 (353) in the experimental group, and using a *t* test for independent samples, a sample size of 22 individuals per group is needed to reject the null hypothesis that the mean levels of ETP are equal in the 2 populations, with a power of 90% and a probability of type I error of 5%.

### Sensitivity Analysis

Different treatment formulations may provide different amounts of testosterone throughout the dosing interval (38). For this reason, a sensitivity analysis was conducted by grouping participants according to the prescribed testosterone formulation (transdermal gel or long-acting intramuscular injection).

## Results

Thirty-eight hypogonadal patients and 38 HCs were enrolled between November 2017 and July 2020. All participants completed the study.

Table 1 summarizes baseline characteristics of hypogonadal patients and HCs. Median BMI was significantly higher in the hypogonadal group compared to HCs. Ten patients presented with primary hypogonadism, 20 secondary hypogonadism, and 8 functional hypogonadism. Except for a lower prevalence of arterial hypertension in patients with primary hypogonadism, no significant differences at baseline were observed among these 3 subgroups (Table 2).

**Table 1.** Characteristics of hypogonadal patients at baseline and of healthy controls

	Hypo (N = 38)	HCs (N = 38)	*P*
Age, y	55 (13)	55 (13)	NS
^a^BMI	28 (26.9-31)	25 (22.9-26.5)	<.0001
^a^BMI classes			
<18.5	0	0	<.001
18.5-24.9	1	14	
25-29.9	20	12	
30-34.9	10	1	
35-39.9	2	1	
>40	2	0	
Diabetes mellitus (n)	3	4	NS
Arterial hypertension (n)	12	14	NS
Dyslipidemia (n)	18	11	NS
Smoker (n)	4	6	NS
Thromboembolic events (n)	0	0	NS
Diagnostic delay, mo	12 (6-25)	NA	—
Fasting plasma glucose, mg/dL	95 (86-104)	NA	—
Total cholesterol, mg/dL	182 (36)	NA	—
Triglycerides, mg/dL	129 (90-206)	NA	—
LDL cholesterol	108 (31)	NA	—

Results are presented as mean (SD) or median (IQR) for quantitative variables, and as absolute frequencies for categorical variables.

Abbreviations: BMI, body mass index; HCs, healthy controls; Hypo, hypogonadal patients; LDL, low-density-lipoprotein; PSA, prostate-specific antigen; N/A, not available; NA, not applicable; NS, not significant.

^*a*^BMI was available for 35 patients and 28 controls.

**Table 2.** Baseline characteristics and coagulation parameters assessed before starting testosterone replacement therapy (T0) in patients with primary, secondary, and functional hypogonadism

	Primary hypogonadism (N = 10)	Secondary hypogonadism (N = 20)	Functional hypogonadism (N = 8)	*P*
Age, y	48 (42-71)	57 (46-66)	62 (41-68)	.95
BMI	28.4 (27.0-30.7)	28.0 (26.8-32.0)	29.0 (26.2-31.7)	.99
Diabetes mellitus (n)	0	2	1	.55
Arterial hypertension (n)	0	8	4	.04
Dyslipidemia (n)	5	8	5	.55
Smoker (n)	0	4	0	.13
Diagnostic delay, mo	18 (5-84)	7 (6-24)	15 (12-24)	.63
Endogenous thrombin potential T0, nM × min	1353 (988-1812)	1849 (1282-1984)	1481 (1068-1724)	.13
Thrombin peak T0, nM	278 (172-377)	376 (262-422)	304 (227-409)	.48
Velocity index T0, nM/min	111 (57-175)	156 (103-181)	126 (91-196)	.56
Lag time T0, min	9.2 (8.5-9.5)	10.0 (9.0-11.1)	9.8 (8.5-10.5)	.15
Time to peak T0, min	11.8 (10.6-12.3)	12.3 (11.4-13.6)	12.0 (10.9-12.8)	.30

Median and interquartile range are reported for continuous variables. Absolute frequencies are reported for categorical variables.

Abbreviation: BMI, body mass index.

Two patients were treated with long-acting injectable testosterone undecanoate, while the remaining 36 received transdermal testosterone 2% gel (median daily dose 30 mg, IQR 20.75-40 mg).

Mean/median testosterone concentrations, hemoglobin, hematocrit, and PSA significantly increased from T0 to T1 (Tables 3 and 4). Testosterone increased from baseline in all patients, and it was above the lower limit of normal (12 nmol/L) in 29 of 38 patients at T1. These parameters remained within safety limits (30) in all patients except 2: in 1 patient PSA increased by more than 1.4 ng/mL (PSA 3.5 ng/mL at T1), while in another patient hematocrit increased more than 54%.

**Table 3.** Changes in testosterone concentrations, complete blood count, and prostate specific antigen in hypogonadal men before (T0) and 6 months after starting testosterone replacement therapy (T1)

	T0	T1	Absolute difference (median)	*P*
Testosterone, nmol/L	6.4 (2.6-8.7)	16.0 (12.5-27.0)	9.9	<.0001
Hemoglobin, g/dL	14.1 (1.1)	15.1 (1.2)	1.1	<.0001
Hematocrit, %	41.7 (3.1)	44.6 (3.3)	2.9	<.0001
PSA, ng/mL	0.55 (0.33-1.03)	0.66 (0.50-1.27)	0.17	<.0001

Results are reported as mean (SD) or median (IQR).

Abbreviation: PSA, prostate-specific antigen.

**Table 4.** Baseline characteristics and coagulation parameters assessed before (T0) and 6 months after starting testosterone treatment (T1), according to testosterone formulation

	Transdermal testosterone 2% gel	Long-acting injectable testosterone undecanoate	*P*
No.	36	2	—
Age, y	58 (27-80)	41 (34-48)	.12
BMI	28 (22-42)	28 (28-28)	.92
Testosterone T0, nmol/L	6.4 (0.1-12.5)	8.0 (5.5-10.4)	.62
Testosterone T1, nmol/L	16.5 (4.3-85.3)	14.1 (12.8-15.5)	.49
Hemoglobin T0, g/dL	14.0 (11.7-17.7)	14.9 (14.6-15.2)	.19
Hemoglobin T1, g/dL	14.8 (12.7-18.1)	16.6 (15.7-17.5)	.11
Hematocrit T0, %	41.2 (36.1-51.4)	43.2 (41.8-44.7)	.39
Hematocrit T1, %	44.0 (38.9-54.6)	47.9 (46.2-49.6)	.10
PSA T0, ng/mL	0.65 (0.00-2.18)	0.47 (0.39-0.55)	.61
PSA T1, ng/mL	0.76 (0.13-3.49)	0.56 (0.56-0.56)	.65
ETP T0, nMxmin	1579 (131-2373)	1857 (1624-2090)	.31
ETP T1, nMxmin	1551 (169-2368)	1438 (1125-1751)	.90
Thrombin peak T0, nM	331 (17-535)	369 (313-426)	.70
Thrombin peak T1, nM	292 (24-542)	279 (201-357)	.67
Velocity index T0, nM/min	137 (3-294)	170 (157-183)	.33
Velocity index T1, nM/min	111 (4-298)	110 (79-140)	.74
Lag time T0, min	9.5 (7.3-13.8)	7.7 (7.3-8.2)	.03
Lag time T1, min	9.5 (7.5-13.5)	8.0 (7.9-8.1)	.07
Time to peak T0, min	12.2 (10.0-17.3)	9.9 (9.7-10.2)	.02
Time to peak T1, min	12.2 (10.2-17.7)	10.5 (10.4-10.7)	.08

Median and minimum to maximum range are reported.

Abbreviations: BMI, body mass index; PSA, prostate-specific antigen.

### Global Coagulation Profile in Hypogonadal Men

No VTE event occurred during the 6-month observation period.

Baseline TGA parameters were not different among primary, secondary, or functional hypogonadism (see Table 2).

**Figure 1.**
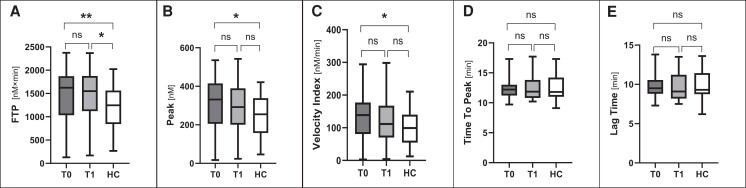
Comparison of thrombin generation assay parameters in hypogonadal men before (T0) and 6 months after starting testosterone replacement therapy (T1), and age-matched healthy controls (HC). A, Endogenous thrombin potential, which accounts for the total amount of thrombin generated in the assay (area under the curve). B, Thrombin peak. C, Velocity index, which results from the following formula: [peak/(time to peak—lag time)]. D, Time to peak, that is, the time (minutes) needed to reach the thrombin peak. E, Lag time, that is, the time (minutes) between the addition of the triggers and the initiation of thrombin generation. ns, not significant; *P less than .05; **P less than .01.

No changes were observed in the whole cohort of hypogonadal men from T0 to T1 in ETP (1502 [536] vs 1485 [512] nM × min; *P* = .77), lag time (*P* = .81), thrombin peak (*P* = .31), time to peak (*P* = .59), and velocity index (*P* = .21) (Fig. 1).

**Figure 2.**
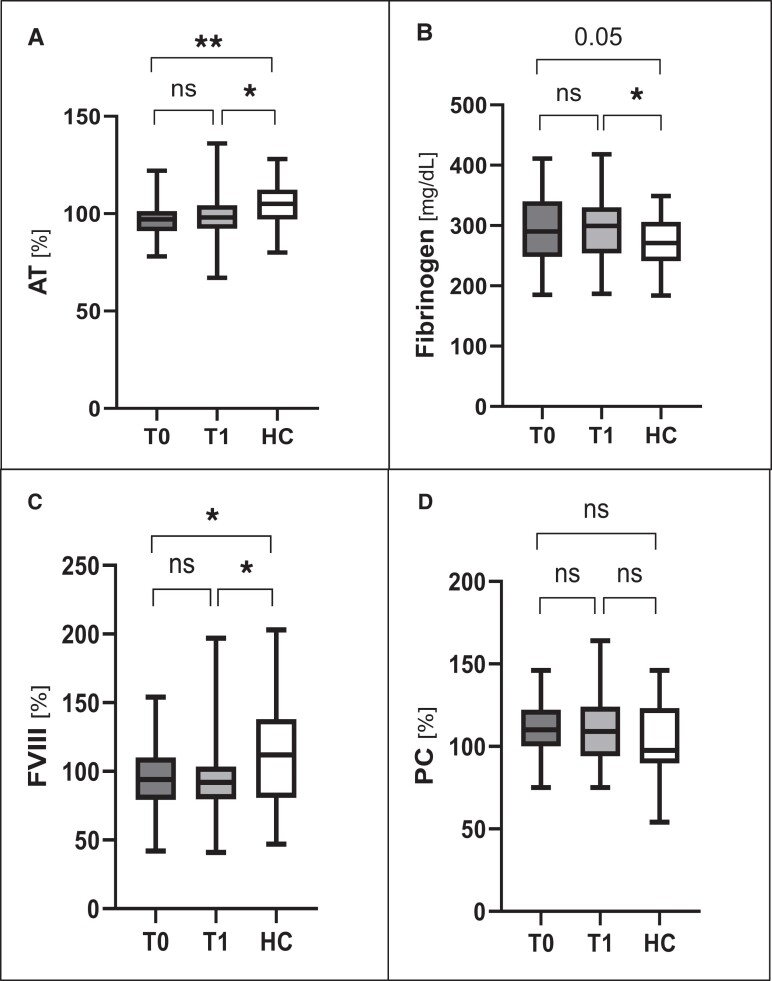
Comparison of other coagulation parameters in hypogonadal men before (T0) and 6 months after starting testosterone replacement therapy (T1), and age-matched healthy controls (HC). A, Antithrombin (AT). B, Fibrinogen. C, Factor VIII (FVIII). D, Protein C (PC). ns, not significant; * *P* less than .05; ** *P* less than .01.

The activity of FVIII, PC, antithrombin, and fibrinogen were comparable between the 2 time points (Fig. 2).

### Sensitivity Analysis

Patients treated with long-acting injectable testosterone were (nonsignificantly) younger than those treated with testosterone gel. With the limitations of small sample sizes (n = 2 and n = 36 respectively), no other significant difference in baseline characteristics was observed (see Table 4).

**Figure 3.**
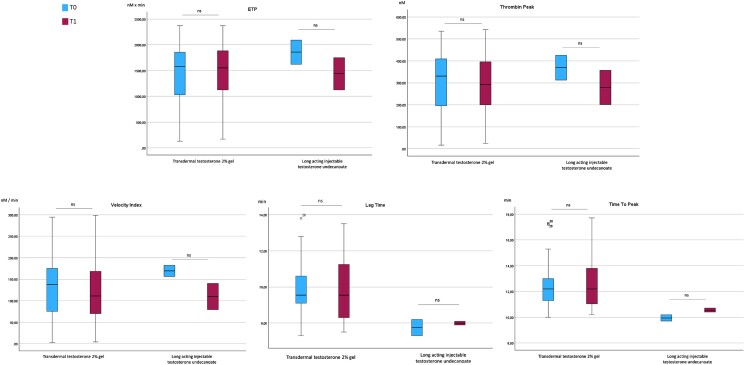
Comparison of coagulation parameters at baseline (T0) and 6 months after starting testosterone treatment (T1), according to testosterone formulation. ETP, endogenous thrombin potential; ns, not significant.

ETP and thrombin peak were comparable between the 2 subgroups at both time points. TGA parameters did not show significant changes from T0 to T1 in either subset of patients (Fig. 3), although, among injectable testosterone-treated individuals, (nonsignificantly) lower ETP, thrombin peak, and velocity index were observed at T1 compared to T0.

### Comparison of the Global Coagulation Profile Between Hypogonadal Men and Controls

Hypogonadal men at baseline displayed significantly increased ETP compared to HCs (1502 [536] vs 1201 [438.1] nM × min; *P* = .009) (see Fig. 1). ETP in patients persisted higher than HCs 6 months after starting TRT (*P* = .01).

The velocity index and thrombin peak showed a significant increase at baseline, but the difference was no longer significant after 6 months, compared to HCs (see Fig. 1). Lag time and time to peak were comparable to HCs (see Fig. 1).

Since hypogonadal men had significantly higher BMI than HCs, analysis was repeated including BMI as a covariate. A significant difference in ETP (*P* = .009), thrombin peak (*P* = .02), and velocity index (*P* = .02) was confirmed after correcting for BMI.

FVIII was lower in hypogonadal men (both at T0 and at T1) than HCs (see Fig. 2). Antithrombin was lower and fibrinogen was higher in hypogonadal patients than in HCs (see Fig. 2). The differences in FVIII, antithrombin, and fibrinogen were no longer statistically significant after controlling by BMI.

### Correlations

No correlation was observed for ETP, antithrombin, and fibrinogen at T0 with age, BMI, diagnostic delay, total cholesterol, triglycerides, or arterial hypertension.

**Figure 4.**
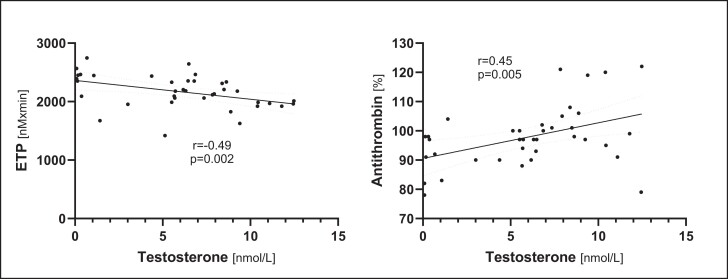
Correlation of basal testosterone concentrations in hypogonadal men with endogenous thrombin potential (ETP, left panel) and antithrombin (right panel). r, Spearman rank correlation coefficient.

Testosterone levels at baseline displayed a significant correlation with ETP (*r* = −0.49; *P* = .002) and antithrombin (*r* = 0.45; *P* = .005) (Fig. 4), but not with fibrinogen.

## Discussion

An increased risk of VTE is observed for women of reproductive age or after menopause on oral contraceptive pills and hormonal replacement therapy (3-5). Whether this risk is increased in hypogonadal men treated with TRT has not been well established (23).

In women, the use of combined hormonal contraception and hormonal replacement therapy has been linked with alterations in plasma levels of nearly all proteins involved in fibrinolysis and coagulation (ie, increases in coagulation factors II, V, VII-XII, and decreases in tissue factor pathway inhibitor and antithrombin levels) (39-41). Therefore, oral contraceptive users have a VTE relative risk ranging from 1.3 to 5.6 depending on the dose of the estrogenic component, estroprogestinic formulation (3, 42, 43), and treatment duration (44), while women on hormonal replacement therapy have a different VTE risk depending on the route of administration of the estrogenic component, oral (odds ratio [OR] 2.5; 95% CI, 1.9-3.4) or transdermal (OR 1.2; 95% CI, 0.9-1.7) (5).

Herein we report that short-term TRT does not affect the coagulation profile of hypogonadal men as measured with a global coagulation procedure that mimics much more than any other coagulation test the process that occurs in vivo. Hypogonadal men display a procoagulant imbalance compared to age-matched HCs, which is correlated with testosterone deficiency but not with hypogonadism subtype (primary, secondary, or functional) or with testosterone formulation administered.

TRT could affect VTE risk leading to an increase in hematocrit, blood viscosity (16), and plasma estrogen concentration (17). In 2015 the US Food and Drug Administration reported a surge in TRT prescriptions, of which 28% to 40% were provided without a biochemical diagnosis of hypogonadism (45). Moreover, based on postmarketing surveillance data, in June 2014 the US Food and Drug Administration and Health Canada issued a labeling change in the product information of all approved TRT formulations regarding the risk of VTE (23). Since then, several clinical trials and metanalyses have investigated the relationship between TRT and VTE risk, reporting conflicting results (18-24, 46-48).

Three meta-analyses of randomized controlled trials documented no association between TRT and VTE risk compared to placebo (22-24). Similarly, 3 observational studies did not detect a significant link between testosterone therapy and risk of VTE (including deep vein thrombosis or pulmonary embolism) in adult men with low testosterone levels compared to hypogonadal men not receiving TRT (46-48).

However, 4 observational studies found an increased VTE risk among patients using testosterone (18-21). Martinez et al and Walker et al demonstrated that this association was most significant within 6 months since therapy start (18, 19).

However, in the study by Martinez et al (18) most patients received TRT following the diagnosis of “nonpathological” hypogonadism based only on clinical data, and in the study by Walker et al (19) only 7.8% of the study population receiving TRT had a biochemical diagnosis of hypogonadism. Furthermore, Kavoussi et al (20) reported an increased risk of deep vein thrombosis only in those hypogonadal patients, diagnosed on the basis of Endocrine Society guidelines, receiving TRT and presenting other potential etiologies for deep venous thrombosis (ie, venous stasis, trauma, genetic disorders). After exclusion of these cases, the overall incidence of thrombosis was similar to the general population.

In the present study, we show that the global coagulation profile of hypogonadal men was not affected by 6-month TRT, and that a procoagulant imbalance is present in this category of patients compared to age-matched HCs. Indeed, some TGA parameters, that is, thrombin peak and velocity index, are significantly higher in hypogonadal men compared to HCs at baseline, but no longer 6 months after starting TRT. This observation may suggest a trend toward the improvement of the procoagulant imbalance following TRT, but a longer time may be needed to achieve normalization. The need for a longer time to attain parameter normalization has already been observed in other endocrine disorders characterized by an increased thrombotic risk, like Cushing's syndrome (37). However, the effects of a longer duration therapy are still to be determined.

We also observed a paradoxical reduction in FVIII levels in the hypogonadal group, which could be regarded as a compensatory mechanism. Testosterone deficiency appears (at least in part) to contribute to this condition, since testosterone levels are inversely correlated with ETP and directly correlated with antithrombin.

Our results are consistent with previously published observations (6-9, 11, 13-15). Low testosterone levels have been correlated with increased platelet activity and a procoagulant profile (increased factor V, VII, X, and fibrinogen and reduced antithrombin) (11, 13). Conversely, both testosterone and dihydrotestosterone have an inhibitory effect on primary hemostasis as measured by in vitro tests, by preventing adenosine diphosphate–mediated platelet aggregation (6). This effect is obtained directly by the activation of a receptor on the platelet membrane, and indirectly through the antiaggregatory effect of nitric oxide produced by the stimulation of endothelial lining cells via the androgen receptor (7, 8). Moreover, testosterone increases the expression of tissue factor pathway inhibitor and tissue plasminogen activator and reduces the secretion of plasminogen activator inhibitor-1, thus inhibiting the coagulation cascade and promoting fibrinolysis (9).

Taken together, these studies suggest that testosterone, at physiological concentrations, has a beneficial influence on hemostasis, thus reducing the risk of VTE. However, 2 population studies have documented no association between endogenous testosterone levels in the lower quartile and new incident cases of VTE compared to individuals with testosterone levels in the mid or upper quartile in age-adjusted analysis (14, 15). These results have been corroborated by a mendelian randomization study that failed to find an association between testosterone concentrations and VTE risk (OR 1.02; 0.74-1.4; *P* = .92; for each 0.1 nmol/L increase in calculated free testosterone) (49). In conclusion, current evidence regarding the association between male hypogonadism and VTE is still scarce and controversial, but suggests that low testosterone levels may actually have a negative effect on the hemostatic system.

It is worth noting that several studies have reported a higher prevalence of thrombophilic disorders (like factor V Leiden or lupus anticoagulant) among patients receiving TRT and experiencing VTE, compared both with individuals with VTE not on TRT (17, 50), and individuals treated with TRT and no thrombotic event (51). In our cohort no patient experienced VTE during the first 6 months of therapy, and participants with previously known thrombophilia were excluded. In this way, our study may have missed investigating a subset of individuals with the highest thrombotic risk. Further studies are needed to explore the effects of testosterone therapy on thrombin generation in the presence of preexisting prothrombotic conditions.

Strengths of this study are the investigation of coagulation of a relatively large and well-characterized group of patients by means of a global assay that takes into consideration much more than any other coagulation test the process that occurs in vivo. There are some limitations, however. First, hypogonadal patients had a considerably higher median BMI compared to HCs, a condition that may be regarded as a potential confounding factor (52-54). Overweight and obesity are considered as comorbidities commonly associated with male hypogonadism and have a higher prevalence in this population (55). On the other hand, components of metabolic syndrome, including visceral adipose tissue, can negatively affect thromboembolic risk (54). Nevertheless, it has been shown that male hypogonadism has a negative effect on hemostasis per se, independently of the association with metabolic syndrome (11-13). Additionally, TGA parameters in our study were still significantly increased in the hypogonadal group compared to the HC group after correcting for BMI, and BMI values showed no correlation with ETP, fibrinogen, or antithrombin. Conversely, testosterone levels had a significant association with ETP and antithrombin. Overall, our results may support a direct role of testosterone deficiency regardless of overweight/obesity. Yet, further studies should compare the coagulation profile in overweight/obese men with and without hypogonadism.

Second, other coagulation factors that influence thrombin generation (eg, tissue factor pathway inhibitor, factor V, protein S, and factor X) (56), platelet activity, endothelial function, and fibrinolysis have not been evaluated in our study. Third, Martinez et al (18) and Glueck et al (50) reported that the incidence of VTE events peaked at 3 months since TRT start. Since in our study intermediate assessments were not performed, any TGA change occurring between 0 and 6 months may have been missed.

In conclusion, this study shows that the procoagulant imbalance observed in hypogonadal men does not worsen following short-term TRT, although robust longitudinal clinical data on the incidence of VTE are lacking. As in the general population, antithrombotic prophylaxis should be warranted in hypogonadal men in case of exposure to other risk factors for venous thrombosis. Further studies are needed to evaluate whether longer-term TRT is able to normalize the procoagulant profile of men with androgen deficiency.

## Data Availability

Some or all data sets generated during and/or analyzed during the current study are not publicly available but are available from the corresponding author on reasonable request.

## Abbreviations

BMI: body mass index
ETP: endogenous thrombin potential
FVIII: factor VIII
HCs: healthy controls
LDL: low-density-lipoprotein
PC: protein C
PSA: prostate-specific antigen
TGA: thrombin generation assay
TRT: testosterone replacement therapy
VTE: venous thromboembolism
